# Assessing cognitive workloads of assembly workers during multi-task switching

**DOI:** 10.1038/s41598-023-43477-0

**Published:** 2023-09-29

**Authors:** Bin Ren, Qinyu Zhou, Jiayu Chen

**Affiliations:** 1 School of Mechatronic Engineering and Automation, Shanghai University, Shanghai, 200444 China; 2 School of Civil Engineering, Tsinghua University, Beijing, 100084 China

**Keywords:** Mechanical engineering, Occupational health

## Abstract

Complex assembly tasks with multiple manual operations and steps often require rapid judgment and action under time pressure and cause most human-related errors. The task switching and action transitions are major sources of these errors. This study intends to implement an electroencephalography (EEG) approach to quantitatively evaluate the mental workload during task switching and transition. The time–frequency and spectrum analysis were utilized to compute and reflect the task demand between the intervals of individual tasks. This study developed an experiment to validate the proposed assessment approach and benchmark the results with the National Aeronautics and Space Administration task load index (NASA-TLX) subjective evaluation scale analysis. The results show that the average value of the power spectral densities (PSDs) of the gamma band signal of the AF4 channel and the beta band signal of Channel F3 show distinctive signal patterns among task stages and intervals. During the interval between the idling stage and the part selection stage, the peak of the PSD envelope increased from 18 to 27 Hz, suggesting advanced cognition increases the mental workload of the interval between different tasks. Therefore, the task switching period cannot be regarded as rest and need to be optimized with better task organization.

## Introduction

Humans play a central role in a complicated and continuous manufacturing process^1^. Manual assembly is more flexible than automated machine assembly and sometimes even costs less time than machinery^2^. Manual assembly usually has multiple and complex task stages, such as the parts selection stage, the tool selection stage, the assembly stage, and the inspection stage, which requires high-level focus and tends to cause errors^3^. Such multi-stage operation usually involves various multi-task switching processes. To understand the impact of multi-task switching on the production process, researchers developed single-task repetitive experiments or multi-task repetitive experiments. Many studies have confirmed the significance of such switching tasks in affecting production efficiency^4^. Researchers also reported that human-related factors are the major consideration for assembly scheduling and labor force planning^5, 6^.

Multi-task switching is more challenging than regular repetitive work as it requires higher human cognitive ability. First, workers have to suffer from time pressure, rapid decision-making, and intensive physical movement. Second, due to temperature, humidity, illumination, noise, etc., the workshop environment often causes discomfort. For example, Golmohammadi et al. reported that even during simple tasks and workers are significantly interrupted by noise, let alone more difficult tasks^7^. Therefore, investigating the impact of cognitive ability, and mental workload, on assembly workers are crucial in improving production efficiency and ensuring the workers’ health. However, physiological conditions cannot be easily monitored onsite and subjective survey results in biased outcomes.

Therefore, it is imperative to implement an effective methodology to accurately assess the cognitive workload experienced by assembly workers. This study aims to proactively address potential emergencies and enhance the longevity of assembly workers' service by employing a wearable electroencephalography (EEG) system. The primary objective is to monitor workers' neural signals, thereby investigating the impact of multi-task switching on cognitive ability and overall job performance. By adopting this approach, the analysis of EEG signals allows for timely early warning notifications to workers. Consequently, managers gain the ability to flexibly and promptly adjust work arrangements, leading to better organization of assembly tasks and a reduction in errors. Furthermore, the optimized work arrangement helps alleviate discomfort and enhances the safety of workshop operations, particularly by addressing issues stemming from excessive workload levels.

## Backgrounds

Task switching is determined to have an impact on mental workload, leading to low working efficiency. Increased task length due to task switching implies reduced productivity, which is an important but not fully discussed aspect of working in safety–critical environments. Walter et al. developed a method to assess the impact of task switching on task completion time and applied it to physician workflows^8^. Zhang et al. investigated whether task switching had an effect on comprehension^9^. The results showed that task-switching behavior has no significant effect on comprehension ability, but it significantly affects the fatigue and workload of the subjects. Clegg et al. explored the effect of task load on the relationship between individual task-switching ability and task engagement^10^. The results showed that the correlation between switching ability and subsequent operation time was only found when there was a high workload in both tasks. In recent years, researchers have also found a strong connection between frequent task switching and negative emotions^11^. For example, Shan et al. explored the rationale for the effects on mental workload and performance after task interruptions. The results showed that the interruption of this task was prone to cause negative emotions^12^. Negative emotion plays a mediating role in the path between task performance and mental workload, which may lead to the increase of mental workload.

In the workshop environment, researchers also used questionnaires and surveys to assess the impact of task switching. In manual assembly tasks, workers are faced with a wide variety of information sources and must switch quickly between different tasks. With proper information representation and planning of work steps, the complexity of task execution can be reduced. For example, information processing during work can be supported by attention guidance, while reducing the number of searches and speeding up assembly execution. Since there are multiple possible assembly orders for a product, the optimal order of the individual assembly steps must be found and the interference of the previous task steps minimized^13^. However, there are different findings. For example, Lu et al. utilized Jersild paradigm to evaluate the effect of task switching between lathe and milling tasks on 18 adult male participants and finally reported no significant difference between the task switching and switching cost (accuracy and response time)^14^. Due to these conflicting findings, the impact of multi-task switching is worthy of further investigation.

**Figure 1:**
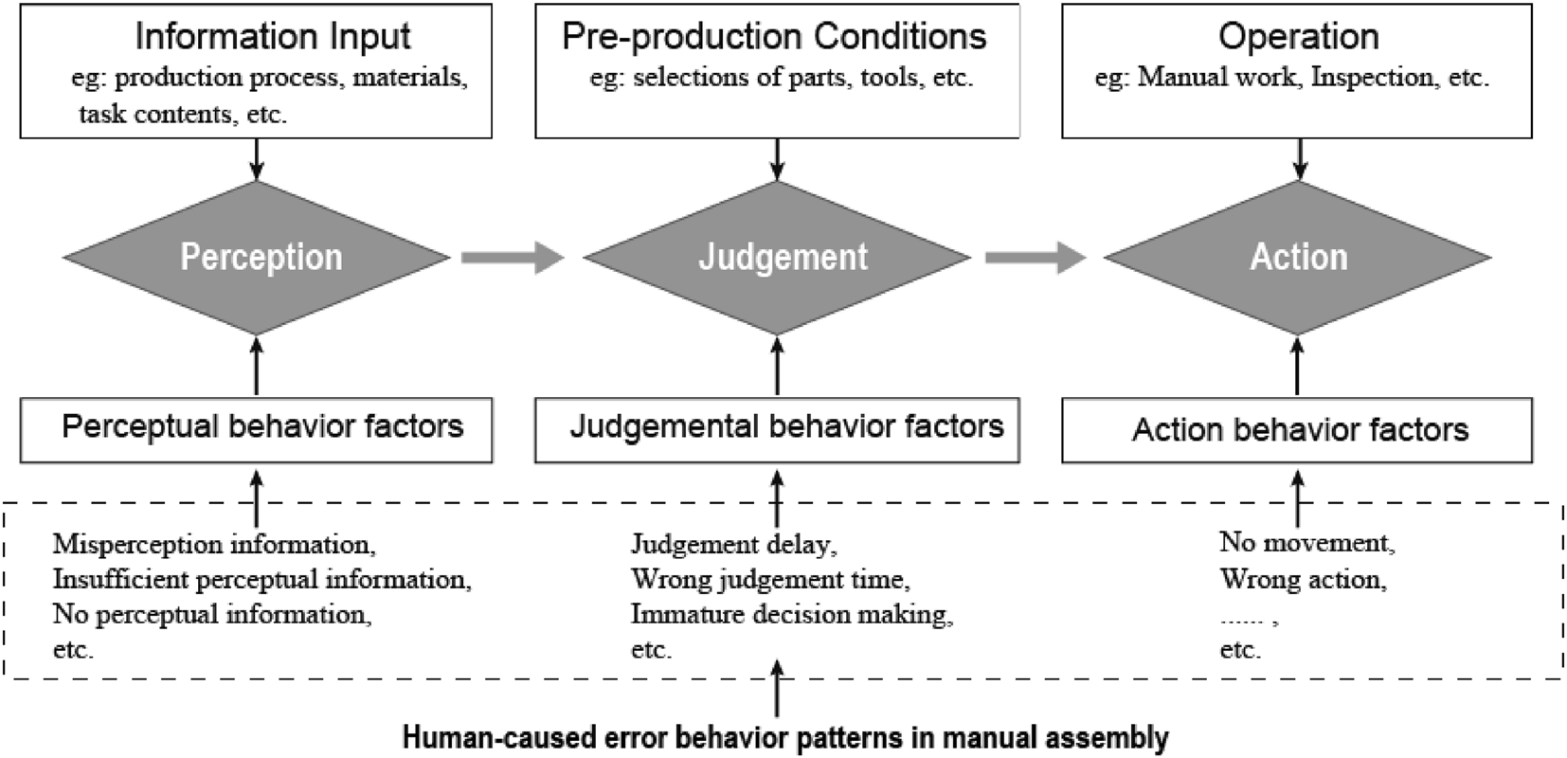
Error model of assembly operators in the workshop.

Errors arising from cognitive overload during assembly tasks can be significantly exacerbated by the interconnected nature of tasks and product components. Chudbery et al. have developed a Stimulus-Organism-Response model (SOR) to comprehensively analyze these errors^15^. Their SOR study suggests that environmental factors can trigger emotional and cognitive responses, leading to either the acceptance or avoidance of potential errors. The SOR model introduces three key elements: external stimulus (S), organism (O), and response (R). When faced with an external stimulus (S), specific psychological activities take place, influencing the state of the organism (O). The organism (O) represents changes within an individual, encompassing emotions, cognition, attitude, and motivation. Building upon the SOR model, this study further identifies three primary types of human errors: perceptual errors, cognitive errors, and behavioral errors, as depicted in Fig. 1. Throughout the entire cognitive process, the mental workload (cognitive load) emerges as a critical resource utilized to complete cognitive tasks and sustain workers' attention across different activities. Hence, this study primarily centers on the quantitative analysis of mental load during task switching, aiming to gain valuable insights into the cognitive demands placed on assembly workers.

### Measurement of task mental load

Compared with measuring the mental load of assembly tasks, the techniques of measuring physical load are more mature and reliable. For example, Lai et al. reported the correlation coefficient between stroke volume variation (SVV) and heart rate variability decreases can be used to quantitatively evaluate whether the body has reached the maximum physical load period^16^. Researchers implemented wearable sensors to capture such high-resolution data and successfully find solutions to prevent work-related musculoskeletal diseases^17^. However, the assessment of mental load is more challenging and still relies on subjective questionnaires^18^. For example, NASA-TLX scales and Borg scales are popular metrics for such mental load and stress assessment^19^. Wang et al. analyzed the NASA-TLX scores with the Technique for Order Performance by Similarity to Ideal Solution (TOPSIS) model and found that the coefficient of variation of the scale was significantly reduced^20^.

In recent years, researchers introduced wearable sensing systems for workshop implementation to capture the physiological status of operators^21, 22^. For example, fatigue states can be detected by monitoring electrocardiogram (ECG) signals, electromyogram (EMG) signals, eye focus movement, etc.^23–25^. Aydemir et al. found that photoplethysmograph (PPG) can replace the traditional subjective techniques for human heart rate detection in practice^26^. Neukirchen et al. used vital capacity equipment to measure the respiratory gases of subjects and found that respiratory gases are sensitive and specific to cognitive load^27^. The respiratory gases have the advantage of predicting cognitive performance and self-reported emotional states and can detect and distinguish different mental needs. In recent years, EEG and fNIRS attracts more attention^28^. With these sensing devices, the researcher can develop more reliable studies for various purposes. For example, Finco et al. studied the rest allowance of workshop operators and optimize the work allocation based on physiological and psychological parameters^29^.

### EEG-based cognitive workload assessment

As a novel tool for understanding cognitive processes, EEG equipment has been widely accepted in recent years. EEG signals have reflective and synthetic signal contents. Usually, EEG signals can be divided into many different frequency bands that are associated with different emotional and cognitive states. Wang et al. reported that delta waves and alpha waves of EEG signals can be used to detect drowsiness^30^. Perna et al. found strong theta wave activity was found in memory-related tasks based on singular paradigms^31^. By comparing gamma waves of EEG signals during motor imagination and actual exercise, scholars found that gamma waves can be used to examine the function of motor imagination neuronal circuits^32^. Some scholars used EEG and electrooculogram (EOG) signals to evaluate users’ vigilance^33^. Fan et al. also employed forehead EEG signals to assess driving fatigue and extended the method for generic fatigue detection^34^. Chen et al. also highlighted that EEG can effectively compute mental loads through gamma-band assessment^35^.

Applying EEG for workshop assembly workers has two major challenges. First, EEG artifacts that are generated by the human body motions can result in assessment errors. Second, the EEG signals are often influenced by electromagnetic devices, eye movement signals, EMG signals, and so on. Therefore, it is necessary to design specific measurement metrics for the manufacturing implementation^36^. The first challenge can be solved with filtering, Independent Component Analysis (ICA), Canonical Correlation Analysis (CCA), etc. To overcome the second challenge, a proper assessment framework is necessary. Wield with these powerful tools, this study intends to assess the cognitive load of multi-task switching based on the EEG signals with the proposed framework. To benchmark the performance of the proposed method, the popular subjective metric NASA-TLX was also adopted to examine the results.

## Methods

### Assessment metric workers’ cognitive workload

**Figure 2:**
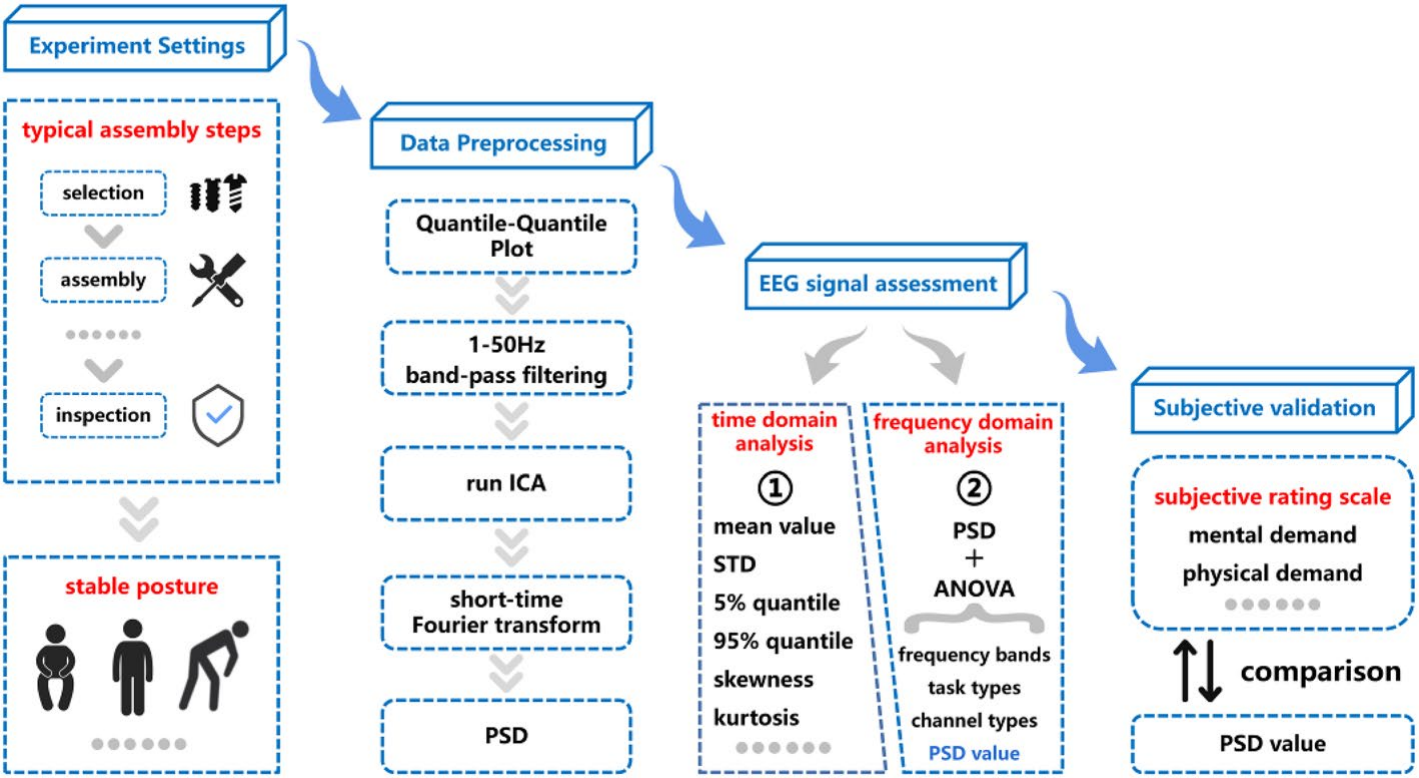
Overview of the assembly workers’ cognitive mental workload assessment metric.

To understand the impact of task switching, this study prepared two groups of tasks, independent tasks, and task intervals. The EEG signals when experiment subjects conduct all tasks are recorded. Figure 2 illustrates the workflow of the mental workload assessment.

Upon the culmination of the experimental phase, a rigorous data preprocessing protocol consisting of five key steps will be meticulously executed. (1) The preprocessing will employ a Normal Quantile–Quantile (Q–Q) plot to ascertain and normalize the EEG signals to an appropriate microvolt level. This step serves to identify and exclude outliers whose data exhibit significant contamination. (2) A band-pass filter spanning from 1 to 50 Hz will be meticulously applied to effectively mitigate low-frequency and high-frequency interference. (3) An Independent Component Analysis (ICA) will be rigorously conducted. This process aids in the identification and removal of irregular data instances, such as those influenced by high-fluctuation eye movement artifacts. (4) A time-domain analysis will be utilized to extract pertinent signal features grounded in sample statistics. This will encompass parameters such as mean, variance, skewness, and kurtosis, facilitating a comprehensive understanding of the data. (5) Frequency-domain analysis will be adopted to delve into the Power Spectral Density (PSD) values of EEG signals across various frequency bands during both independent tasks and task intervals, including task switching. This holistic methodology will yield valuable insights into the frequency distribution and inherent characteristics of EEG signals under diverse cognitive demands.

Finally, as part of an external evaluation, the post-experiment NASA-TLX scale will be employed to assess the cognitive workload experienced by the assembly workers during the experimental tasks. This assessment will offer a standardized and objective measure of the perceived cognitive demand, further enhancing the comprehensiveness of the study.

### Experiment design

#### Experiment apparatus

**Figure 3:**
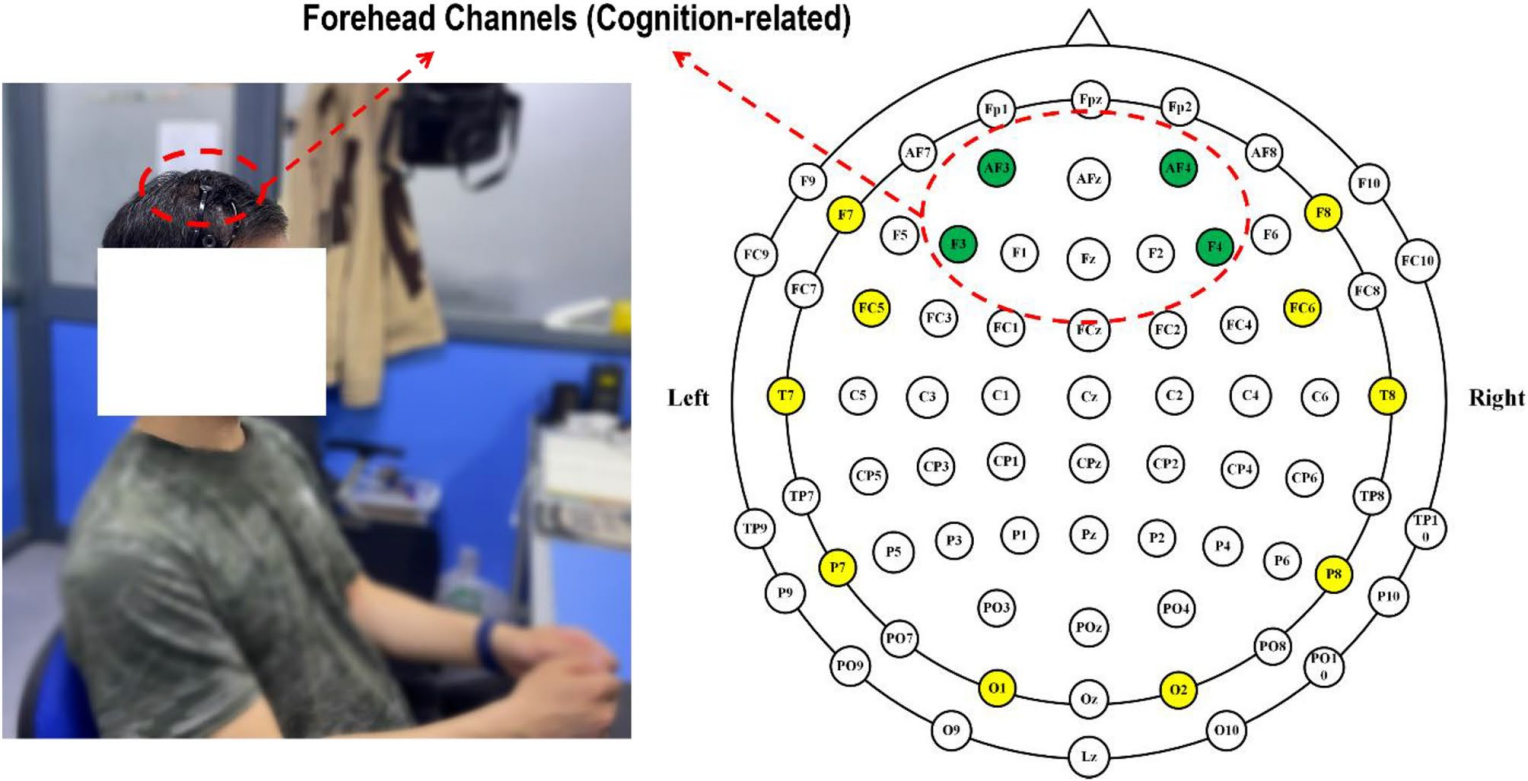
Emotiv Epoc+ and the forehead channels (AF3, F3, F4, and AF4).

This study utilized Emotiv Epoc+ for EEG signals collection with a sampling frequency of 256 Hz. Before the experiment, the device electrodes need to be completely wet with saline to maintain good contact with the scalp. The Emotiv Epoc+ has 14 channels (Fig. 3). The forehead region is proved to be more related to cognition^35^. Therefore, as shown in Fig. 3, the channels marked in green are the major signal acquisition channels (AF3, F3, F4, and AF4), as they have less hair coverage and are highly related to cognition activities.

#### Experiment settings

The independent tasks include four stages, the idling stage, selection stage, assembly stage, and inspection stage. The switching tasks are the intervals between independent tasks. All subjects are required to seat during the idling stage, squat during the assembly stage, seat while inspection, and stand during task intervals. This setup aims to replicate the working posture and working conditions of the actual assembly workers in the workshop. The idling stage required participants seat and rest for three minutes. In the selection and assembly stage, the subjects were asked to select standard parts and assembly tools for a prosthetic knee mechanism. In the assembly stage, the subjects have to keep squatting to complete the whole assembly task to mimic the physical load in the actual assembly work. The inspection stage required the subjects to measure the dimensions of the key parts of the prosthetic knee joint mechanism and check whether the assembled product met the requirements of freedom of movement.

**Figure 4:**
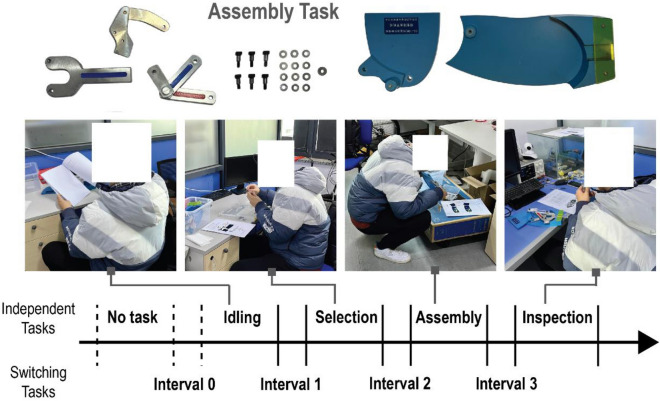
Experiment process and task organizations.

This study comprised a cohort of 29 undergraduate students, aged between 19 and 21 years, with 27 male students and 2 female students. Prior to the experiment, all participants underwent assembly training to ensure a basic level of familiarity with the tasks. A comprehensive overview of the entire experiment process is presented in Fig. 4. Following the experimental tasks, the recruited 29 students were required to complete the NASA-TLX scale. This survey incorporates two distinct sets of scores—weight scores and rating scores. Weight scores are utilized to gauge the relative impact of the workload imposed by each task, while rating scores provide valuable insights into the subjects' opinions on the influence of individual contributing factors. By utilizing this well-recognized assessment tool, the study effectively captures and quantifies the perceived cognitive workload experienced by the participants during the experimental procedures.

### Ethical approval

The experiment involved in this article has been reviewed and approved by ethics committees of Shanghai University. All participants information are eliminated for protection of privacy. The development of experiments follows the regulation of the Ministry of Civil Affairs of the People’s Republic of China, National Research Center for Rehabilitation Technical Aids and China Rehabilitation Research Center (http://kffj.mca.gov.cn/ and https://www.crrc.com.cn/). All experimental protocols were approved by the academic ethics committee of Shanghai University. All methods were carried out in accordance with relevant guidelines and regulations. Informed consent was obtained from all subjects. The following is the short informed consent statement, the full statement can be found in Appendix A.

### Informed consent

I have been informed of the purpose, background, process, risks, and benefits of the study.

I have ample time and opportunity to ask questions, and I am satisfied with the answers.

I have read this informed consent form and agree to participate in this study.

I understand that I can choose not to participate in the study or withdraw from the study at any time during the study without any reason”.

## Results

### Independent tasks

#### Time-domain analysis

**Figure 5:**
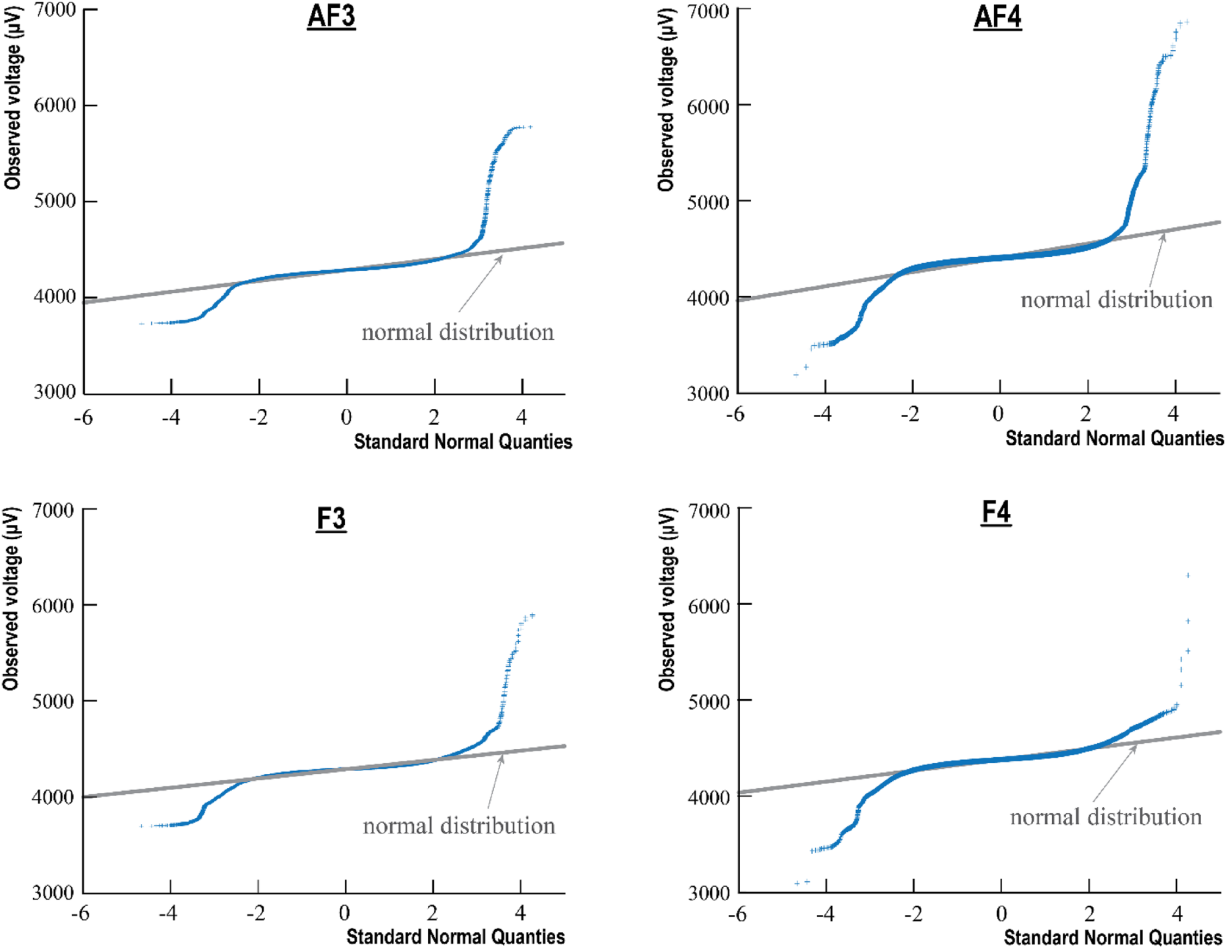
Q–Q plot and power spectrum of a single subject.

Following Fig. 5 shows the Q–Q plots of all channels of a single subject. As it can be seen that the performance of the EEG signal, most of the sample points follow the standardized normal distribution, which suggests the acquired signals are stable and reliable during the experiment. Then, the qualified data will be filtered by a 1–50 Hz band-pass signal filtering and spikes caused by artifacts will also be removed with the ICA algorithm.

Table 1 shows the time-domain data statistics of all 29 subjects at each stage. The average value reflects the general level of EEG sample points; Standard deviation (STD) reflects the dispersion of a data set and it can be used to measure the amount of fluctuation in a sample. The greater the standard deviation, the greater the volatility of the sample data. Quantile refers to the data value of the sample points on the corresponding percentile after the sample points are sorted from small to large. Skewness can judge the degree of asymmetry of data distribution, and kurtosis can study whether data distribution is smooth.

**Table 1:** Summary of EEG Data Statistics.

Channel	Mean ($\mathrm{\mu V}$)	STD ($\mathrm{\mu V}$)	5% quantile ($\mathrm{\mu V}$)	95% quantile ($\mu V$)	Skewness	Kurtosis
*Activity-idling*						
AF3	4186.71	120.47	4068.58	4340.89	− 0.00	27.52
F3	4187.18	99.48	4096.92	4318.84	− 0.67	17.37
F4	4218.89	157.24	4083.46	4425.76	1.88	46.42
AF4	4231.31	173.12	4056.02	4470.38	0.75	8.66
*Activity-selection*						
AF3	4197.78	169.33	4016.28	4417.82	− 1.49	41.74
F3	4195.34	109.11	4088.97	4347.82	− 0.56	16.41
F4	4232.08	164.66	4066.66	4490.76	0.32	6.01
AF4	4245.07	220.40	4009.61	4571.79	1.44	10.42
*Activity-assembly*						
AF3	4187.87	197.08	3971.53	4437.69	0.82	23.28
F3	4187.77	112.73	4063.46	4353.71	− 0.69	15.78
F4	4219.78	173.07	4047.82	4476.28	1.67	32.16
AF4	4228.46	235.45	3950.89	4584.23	0.85	10.24
*Activity-inspection*						
AF3	4198.89	125.17	4060.25	4353.84	0.69	35.22
F3	4198.69	100.09	4093.46	4333.33	− 0.59	11.63
F4	4235.88	141.25	4084.87	4456.92	0.48	3.49
AF4	4246.20	185.75	4047.05	4503.07	1.23	31.38

From the time-domain data (Table 1), we can find that the mean value and STD of voltage data in the AF4 channel are in the lead, indicating that the data in the AF4 channel is highly discrete, which is beneficial to distinguish task types. The skewness values of the sample points of each channel in each task type basically remain around 0, indicating that the sample points of each channel are relatively close to normal distribution. The kurtosis value of the sample points in each channel is greater than 0, so the data distribution curves of the sample points in each channel under different task types tend to be "sharp", indicating that there are outliers on both sides of the curve, which can be intuitively observed from the Q–Q plot. Based on the kurtosis characteristics of sample points in each channel, it can be concluded that the data distribution curve of the AF4 channel is closer to the normal distribution in the idling stage and assembly stage, and the data distribution curve of the F4 channel is closer to the normal distribution in the parts selection stage and inspection stage. By setting the 5% quantile and the 95% quantile, we can correct for outliers with large deviations.

#### Frequency-domain analysis

**Figure 6:**
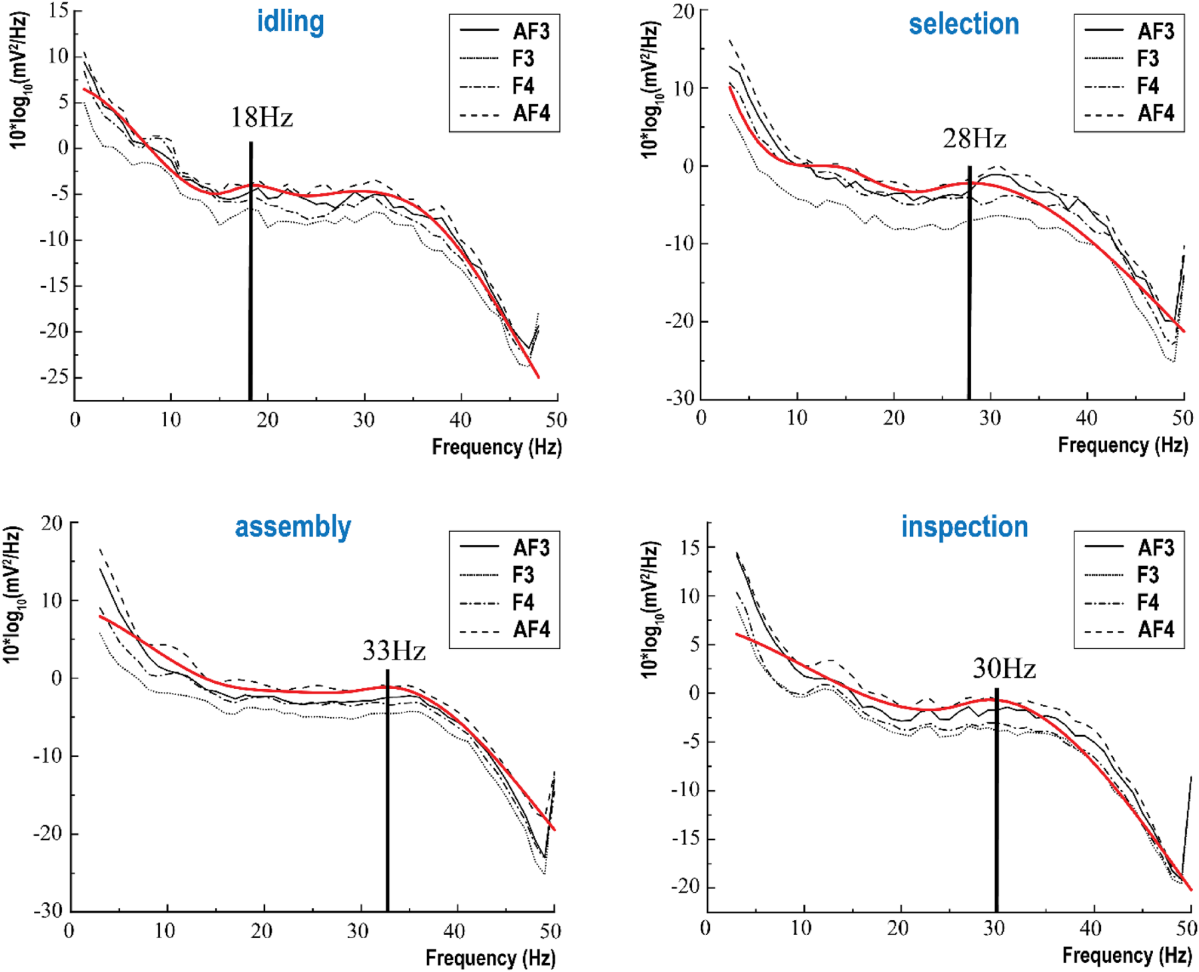
Power spectrum intensity and peak identification of power spectrum for all subjects.

Figure 6 shows the overall task power distribution of four channels across the frequency spectrum. The red curve traces the PSD envelope curve of the AF4 channel which is closely correlated to the frequency spike of activity. By tracing the peak position of the envelope curve, we can distinguish the different stages of the whole task. For example, in the idling stage, there are obvious peaks at 18 Hz. The 18 Hz band is a typical beta wave, which is related to thinking analysis, alertness, and tension. At the selection stage, two peaks were observed. The first peak still appeared in the beta band and the second peak occurred in the 28 Hz band. The frequency band of 28 Hz is very close to the gamma wave, indicating that the subjects have gradually entered a cognitive state when choosing parts and tools. In the assembly stage, an obvious peak appeared in the frequency band of 33 Hz. 33 Hz belongs to the gamma wave, which is related to problem-solving, learning and cognition, and coping with challenges, indicating that the subjects entered a deep state of cognition and learning during the prosthetic knee joint assembly. In the final inspection stage, a peak appeared at the 30 Hz band. However, compared with the assembly stage, the cognitive load in the inspection stage is reduced.

**Figure 7:**
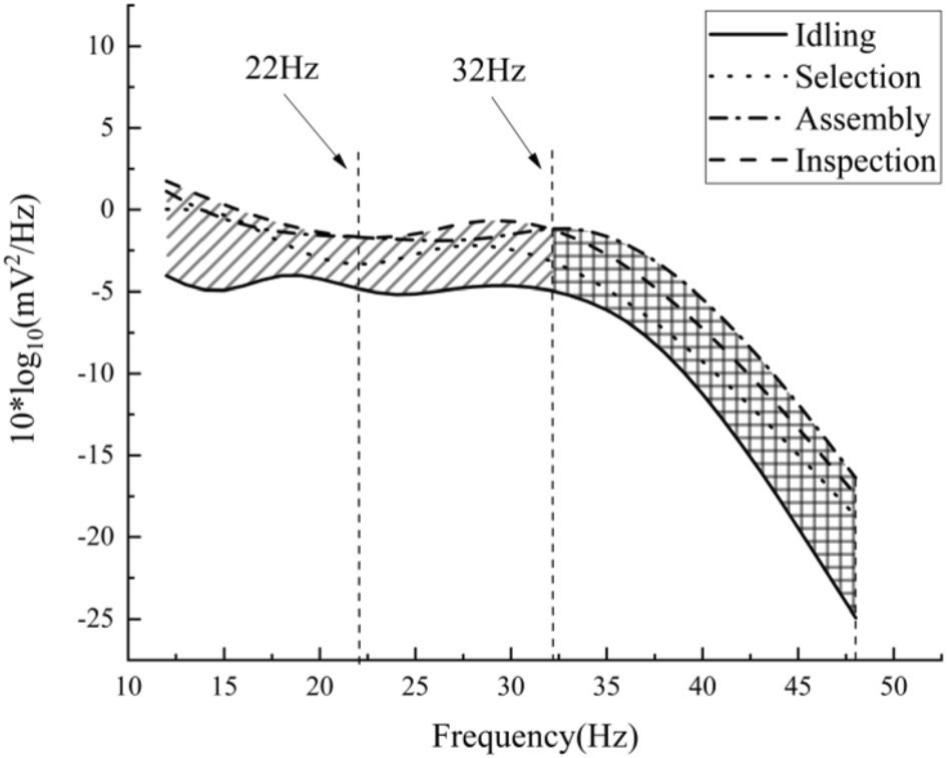
Envelope diagrams of four task stages.

Figure 7 shows the boundaries of all four envelope curves. Taking the idling stage as the baseline, the area enclosed by the curves suggests the mental load increases. The maximum PSD appears at 32 Hz, indicating that the EEG signal power spectrum of the subjects in the inspection stage varied most in the beta band. After 32 Hz, the PSD envelope curve shows the highest difference compared with the baseline appearing at the gamma band.

To sum up, it can be concluded that the method of distinguishing different stages in assembly tasks based on the PSD graph is valid and effective. When the cognitive demands of the subjects increased, the peak in the PSD graph would be closer to the gamma band, and the overall PSD value of the gamma band would also increase. Therefore, through the test of gamma-band PSD value, different tasks’ demands can reflect the level of the mental load of subjects.

#### Variance analysis

A three-factor ANOVA was conducted to explore the correlations between task, channel, and channels and verify if these variables have distinctive patterns. All examined factors are defined as categorical variables. The band variables are PSD valued that grouped by typical band definitions for the alpha band (8–12 Hz), the beta band (13–30 Hz), and the gamma band (31–50 Hz). Table 2 summarizes the results of three-factor ANOVA. It can be concluded that task types (P = $3.89\times {10}^{-7}$ < 0.01 < 0.05), channel types (P = $4.64\times {10}^{-11}$ < 0.01 < 0.05), and frequency bands (P = 0 < 0.01 < 0.05) have significant impact on final PSD values. The P values of the Task*Channel (P = 0.78 > 0.05), Task*Band (P = 0.74 > 0.05), Channel*Band (P = 0.99 > 0.05), and Task*Channel*Band (P = 1 > 0.05) indicate that there are no obvious correlations among all three categorical variables.

**Table 2:** Three-factor analysis of variance results.

	DF	SS	MS	F	p	${\eta }_{p}^{2}$
Task	3	600.26	200.08	11.14	3.89E10−7	0.050
Channel	3	954.48	318.16	17.72	4.64E10−11	0.077
Band	2	9151.75	4575.87	254.85	0.00	0.443
Task*Channel	9	98.63	10.95	0.61	0.78	0.009
Task*Band	6	62.55	10.42	0.58	0.74	0.005
Channel*Band	6	13.59	2.26	0.12	0.99	0.001
Task*Channel*Band	18	18.01	1.00	0.05	1.00	0.002
Model	47	11,780.97	250.65	13.96	0.00	0.000
Error	640	11,491.15	17.95	0.00	0.00	0.000
Revised overall	687	23,272.12	0.00	0.00	0.00	0.000

*DF* degree of freedom, *SS* the sum of squares of deviation from mean, *MS* mean square, ${\eta }_{p}^{2}$ partial eta squared.

Table 3 shows the group variance of all independent variables. Group variables of most categorical groups are similar, except for the gamma band. This suggests that the PSD intensity of the gamma band can be a good indication to represent mental load features.

**Table 3:** PSD variance of different variable category groups.

Variable	Category	Group variance
*Task*	Idling	33.77
	Selection	33.18
	Assembly	33.76
	Inspection	28.75
*Channel*	AF3	31.24
	F3	31.25
	F4	32.72
	AF4	33.38
*Band*	Alpha	4.20
	Beta	5.05
	Gamma	31.54

#### NASA-TLX validation

**Figure 8:**
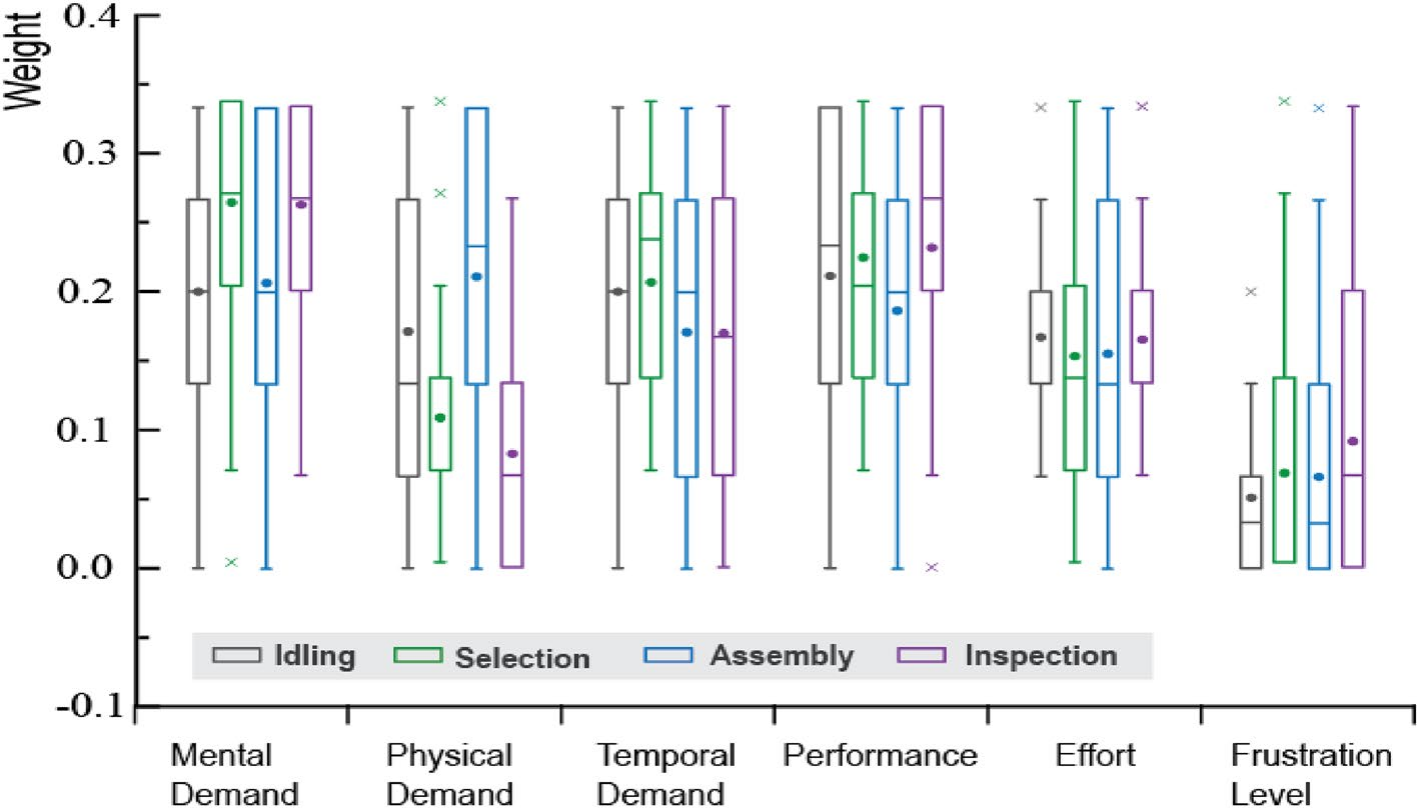
Weight box diagram of six indicators of the NASA-TLX scale.

This study slightly adjusted the NASA-TLX, so that the items are understandable to all subjects. The original NASA-TLX scale scores have 21 positive integer scores from 0 to 100 with an interval of 5. The adjusted NASA-TLX scales still have 21 intervals, but introduced negative scores. This setup aims to reflect the subjects’ feelings of difficulty with the work—the “acceptable” (low difficulty) options ranged between − 1 to − 10 and the “unacceptable” (high difficulty) options ranged between 1 and 10. Figure 8 shows the box plots of NASA-TLX indicators’ weights for all four tasks. As shown in the Figure, the part selection stage has the highest mental load and lowest physical load. The manual assembly stage has both demands at a high level.

**Figure 9:**
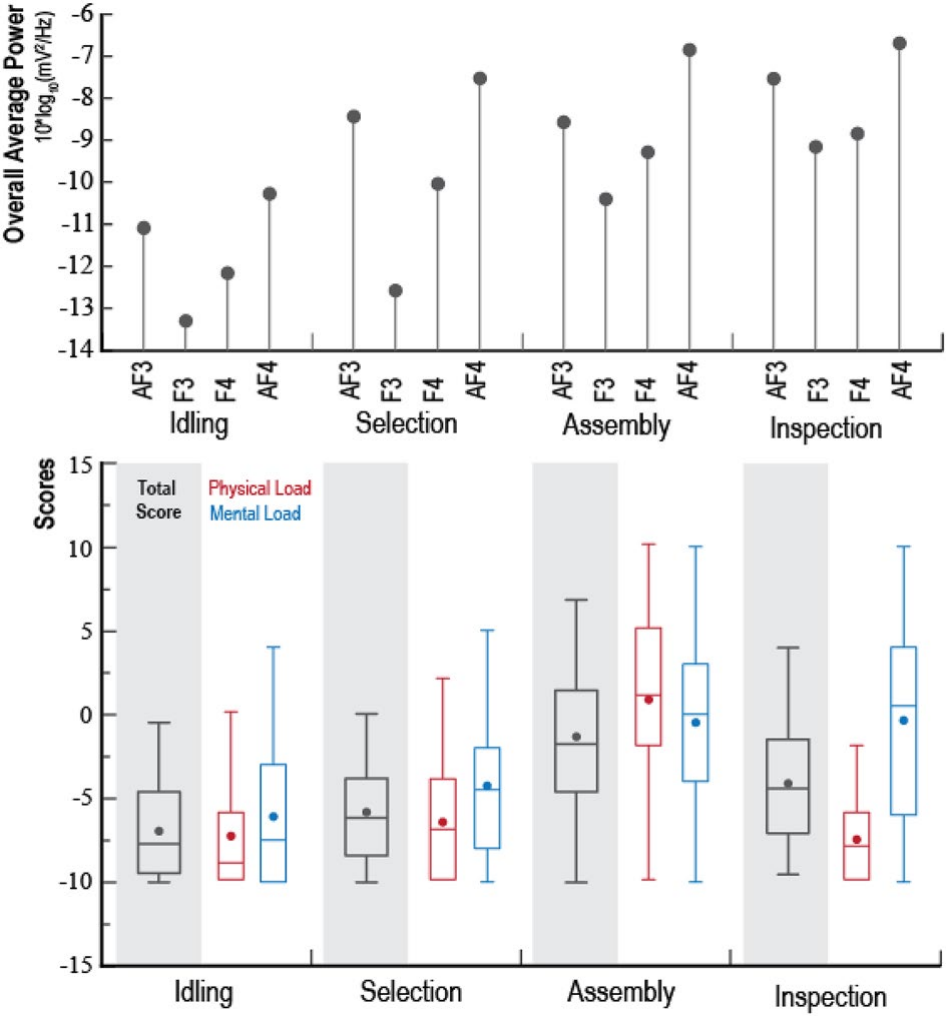
The total mean power of the gamma band and NASA-TLX scale score.

Figure 9 compares the assessed PSD and NASA-TLX scores. The NASA-TLX scores in general match the trend of the computed mental load of gamma band signals. The AF4 channel has peaked in the assembly stage and the inspection stage, which is identical to the mental load scores of the NASA-TLX scale.

### Switching task intervals

#### Time-frequency analysis

When workers switch tasks, they tend to lose their concentration on the current task. It is likely to assume workers may turn into a relaxed state and reduces their mental load. However, except for rest and short breaks, workers are not in a relaxed state in actual task switching. They may still retain the cognition state of the previous task and immerse in the preparation of the next task. Such residual and advanced cognition states cannot be directly reflected in the subjective rating scales, but they can be captured with continuous EEG monitoring.

To analyze such task switching state, this study composed three virtual “tasks”, including Interval 1 (between idle and selection stage), Interval 2 (between selection and assembly stage), and Interval 3 (between assembly and inspection stage). Table 4 shows the data statistics of all virtual “tasks”. The average time of the interval from the idling stage to the part selection stage is ${\mathrm{T}}_{\mathrm{i}\left(1\right)}=34.78\mathrm{ s}$. The average time of the interval from the part selection stage to the assembly stage is ${\mathrm{T}}_{i\left(2\right)}=26.56\mathrm{ s}$. The average time of the interval from the assembly stage to the inspection stage is ${T}_{i\left(n\right)}=48.81\mathrm{ s}$.

**Table 4:** Summary of EEG data statistics during task switching.

Channel	Mean	STD	5%Q	95%Q	Skewness	Kurtosis
*Interval 1*						
AF3	4224.98	125.46	4073.88	4428.21	2.88	16.56
F3	4228.32	87.50	4137.44	4398.85	0.54	2.71
F4	4276.81	151.44	4130.90	4624.87	1.07	3.69
AF4	4309.97	217.18	4080.02	4650.32	2.34	16.90
*Interval 2*						
AF3	4215.70	77.99	4109.91	4381.41	0.79	2.12
F3	4211.97	130.69	4135.83	4351.99	− 5.57	96.17
F4	4245.79	137.27	4037.50	4495.13	1.95	9.41
AF4	4239.77	238.22	3826.95	4582.18	0.74	7.14
*Interval 3*						
AF3	4173.44	61.17	4080.86	4262.83	0.64	4.11
F3	4175.15	36.52	4119.68	4225.64	0.13	3.67
F4	4201.81	43.06	4130.58	4273.48	− 0.07	− 0.42
AF4	4205.23	62.36	4103.21	4300.32	0.33	1.25

**Figure 10:**
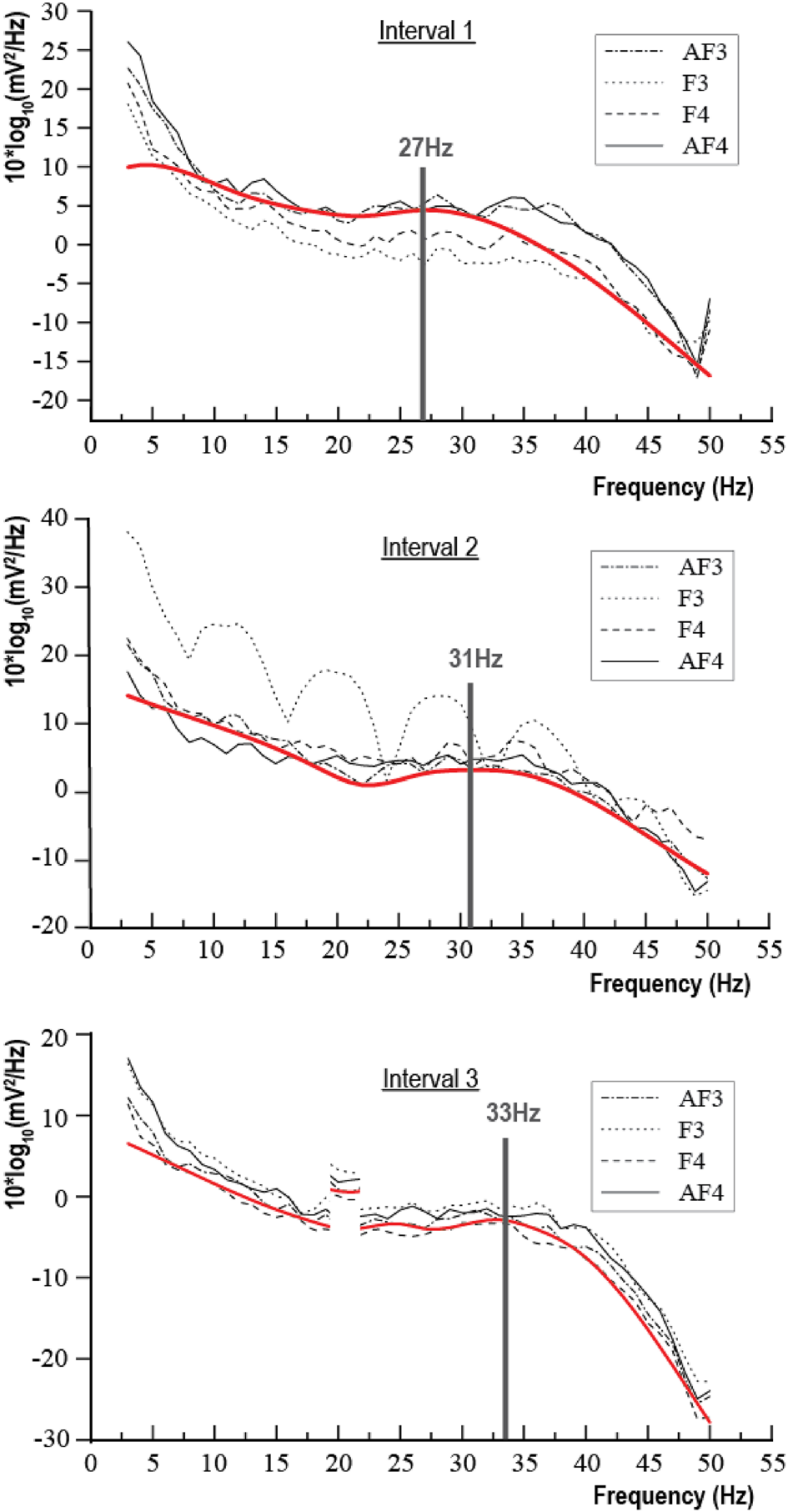
EEG PSD of all subjects during multi-task switching.

Figure 10 displays the PSD diagram of EEG signals observed across all three intervals. Evidently, the peak values within these intervals exhibit a discernible pattern in comparison to the idle stage, as depicted in Figure 6 (18 Hz). Notably, the power distribution pattern tends to exhibit similarities with the subsequent or preceding stages. For instance, considering Interval 3, its preceding stage is the assembly stage, while its subsequent stage is the inspection stage. In Interval 3, a wave peak is observed at 33 Hz, closely resembling that of the assembly stage (Fig. 6). These findings suggest a consistent trend in the power distribution pattern across consecutive stages, possibly indicating underlying cognitive processes and workload variations during different task intervals.

#### Variance analysis

Similar to tasks, the intervals also can be analyzed with a univariate three-factor ANOVA to investigate its major influencing factors. The examined variables include the interval type, channel, and band group. Table 5 lists the results of the ANOVA test. The item Interval*Channel*Band has a P value of 0.04, indicating that there are statistically significant interactions among interval type, channel, and band group.

**Table 5:** Analysis results of the three-factor ANOVA.

	DF	SS	MS	F	P	${\eta }_{p}^{2}$
Interval	2	2751.35	1375.68	56.03	0.00	0.189
Channel	3	255.78	85.26	3.47	0.02	0.021
Band	2	10,309.22	5154.61	209.94	0.00	0.467
Interval*Channel	6	2103.21	350.53	14.28	5.33E−15	0.151
Interval*Band	4	211.37	52.84	2.15	0.07	0.018
Channel*Band	6	343.11	57.18	2.33	0.03	0.028
Interval*Channel*Band	12	550.91	45.91	1.87	0.04	0.045
Model	35	17,761.53	507.47	20.67	0.00	0.000
Error	480	11,785.33	24.55	0.00	0.00	0.000
Revised overall	515	29,546.86	0.00	0.00	0.00	0.000

Then simple effects analysis is conducted in Table 6. Table 6 covers the interrelation test for different channels. Comparing across all channels, it is worthy to notice that the Interval*Band has a statistically significant p value for the F3 channel but insignificant p values for all other channels.

**Table 6:** Interrelation test between interval and band across all channels.

	DF	SS	MS	F	P
*AF3*					
Interval	2	551.40	275.70	10.63	5.59E−05
Band	2	1860.74	930.37	35.89	6.06E−13
Interval*Band	4	26.36	6.59	0.25	0.90
Model	8	2846.82	355.85	13.72	5.34E−14
Error	120	3110.63	25.92	–	–
Revised overall	128	5957.46	–	–	–
*F3*					
Interval	2	3400.08	1700.04	57.60	0.00
Band	2	4282.72	2141.36	72.56	0.00
Interval*Band	4	693.96	173.49	5.87	2.35E−04
Model	8	8504.66	1063.08	36.02	0.00
Error	120	3541.35	29.51	–	–
Revised overall	128	12,046.01	–	–	–
*F4*					
Interval	2	393.17	196.58	8.90	2.48E−04
Band	2	2616.46	1308.23	59.26	0.00
Interval*Band	4	15.20	3.80	0.17	0.95
Model	8	3198.67	399.83	18.11	0.00
Error	120	2648.90	22.07	–	–
Revised overall	128	5847.58	–	–	–
*AF4*					
Interval	2	509.89	254.94	12.31	1.37E−05
Band	2	1892.39	946.19	45.70	1.78E−15
Interval*Band	4	26.74	6.68	0.32	0.86
Model	8	2819.84	352.48	17.02	1.11E−16
Error	120	2484.43	20.70	–	–
Revised overall	128	5304.27	–	–	–

Based on the above observation, this study also conducted band group variance analysis, especially for the F3 channel. The results show that the PSD group variances for the alpha band (around 2.51–2.61) and gamma band (1.31–2.15) are relatively weak. The beta band shows significant variance differences for all intervals (Interval 1 is 5.92, Interval 2 is 27.34, and Interval 3 is 76.47). Therefore, the PSD variation of the beta band on the F3 channel can be used to distinguish the task switching intervals.

#### Cross-task summary

**Figure 11:**
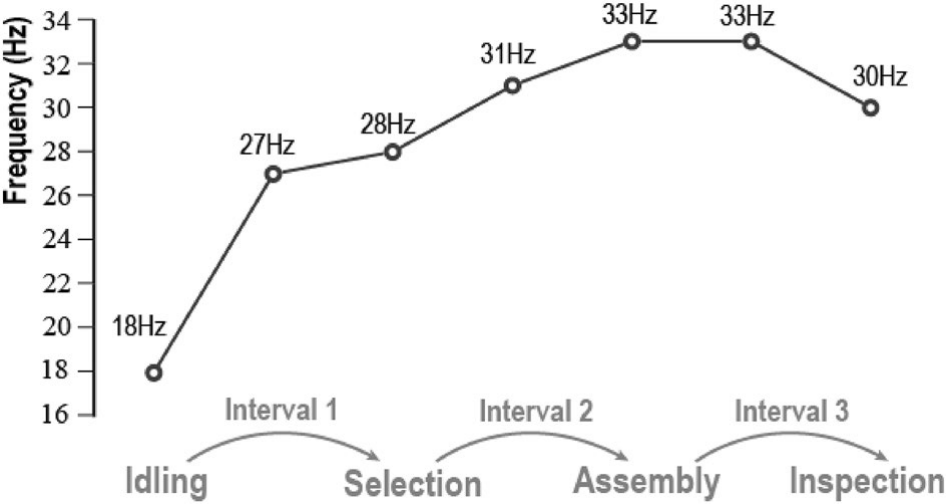
Peak frequency variation of the whole assembly task.

The PSD peak of the power distribution envelope can be a possible indicator of the mental state of workers. Its intensity and event of exceeding the threshold can be used to reflect different mental loads, task switching efficiency, and the possibility of errors. Figure 11 shows the transition of the peak frequency of PSD for all tasks. The peak frequency is usually between the previous and succession tasks of a task switching interval. The corresponding peak frequency of intervals appears in the beta band range and is different from the idling stage.

In Interval 1, the preceding stage is the idling stage, while the subsequent stage is the selection stage. Notably, the PSD peak of Interval 1 exhibits a substantial increase when compared to the idling stage. However, it closely approximates the PSD peak observed during the selection stage, suggesting a precognitive state among the subjects. Similarly, in Interval 2, the preceding stage is the selection stage, and the subsequent stage is the assembly stage. The PSD peak of Interval 2 indicates that the subjects do not experience a notable relief from the demands of the selection task, as they seem to be already cognitively engaged in anticipation of the upcoming assembly task. Moving on to Interval 3, the preceding stage is the assembly stage, followed by the inspection stage. The PSD peak of Interval 3 reveals that the subjects sustain a heightened level of cognitive load, likely due to the lingering impact of the assembly task. In this interval, their mental workload does not dissipate significantly, further highlighting the continuous cognitive demand placed on the subjects during this phase of the experiment.

## Discussion

Assessing workers' cognitive ability through the use of EEG devices presents a potent approach for investigating the mental load associated with assembly tasks and transitions between them. The experimental findings indicate a direct correlation between the gamma band of the AF4 channel and variations in workers' mental load. Additionally, distinctive patterns are observed in the beta band of the F3 channel during task transitions, reinforcing the connection between cognition and the forehead region, which aligns with the research conclusions of Takahashi et al. and Wang et al.^37, 38^. Siddiquee et al. also noted significant cognitive workload classification accuracy when focusing on the mid-forehead location compared to using whole forehead sensing^39^. It is noteworthy that in this study, the AF4 channel from the right forehead demonstrated a high correlation with cognitive workload, a noteworthy finding not previously reported in Siddiquee's research. Furthermore, the investigation highlights the association of the gamma band with cognition during the assembly process, wherein manual operations are involved, consistent with the findings of Xiao et al.^40^. Moreover, other researchers have suggested that the alpha band and theta band are linked to cognitive overload across tasks^41^. These conclusive outcomes underscore the viability of employing mental load assessment to explore task requirements and transitions effectively. The study's results contribute valuable insights into the cognitive dynamics of assembly tasks, paving the way for enhanced understanding and optimization of cognitive performance in a workplace context.

The manufacturing industry operates within a fiercely competitive market, characterized by increasing client demands. Manual assembly workers, in particular, confront complex task information and tight schedules, leading to elevated levels of mental fatigue and error rates^42^. Practical experience demonstrates that errors in assembly tasks often arise from overloaded recognition capacity and improper transitions during operations. The experiment's findings reveal that, even after completing a task stage, the mental load remains sustained. This suggests that task transitions cannot be treated as periods of rest and continuously consume workers' cognitive capacity, increasing the likelihood of operational errors. As a result, these transitions fail to adequately meet the workers' need for rest. Abdous et al. have previously proposed a quantitative fatigue and recovery criterion to prevent individuals from entering an overloaded conditionsss^43^. Moreover, the level of mental load during task transitions exhibits a strong association with the preceding and succeeding stages. The proposed task switching assessment method assumes a pivotal role in optimizing task organization and work design. By employing quantitative mental load assessment, the optimized task arrangement can incorporate necessary periods of rest during task transitions, ultimately reducing the overall cognitive burden throughout the task process. This, in turn, mitigates the possibility of rework and enhances overall efficiency. Furthermore, the proposed methods offer the additional advantage of evaluating the efficacy of work allowance aimed at alleviating worker fatigue. Studies by Sharpe et al. have indicated that interventions targeting cognition through EEG signals, such as binaural beat psychoacoustic stimulation at 40 Hz (gamma band), can yield improvements in cognition, memory, and mood^44^. Therefore, the proposed method can be extended to investigate intervention approaches, further enhancing work safety and efficiency. These interventions hold the potential to elevate worker well-being and overall performance within the demanding manufacturing environment.

This study, while providing valuable insights, does exhibit certain limitations that warrant consideration and resolution in future research endeavors. Firstly, during the data preprocessing stage, the ICA method was employed to address electric artifacts caused by eye blinking. Nevertheless, the removal of abnormal artifacts necessitated manual intervention, heavily relying on the expertise of researchers. To enhance practical usability, the development of automated data processing techniques becomes imperative. A promising avenue for future investigations lies in the exploration of more robust wavelet analysis approaches capable of effectively eliminating data contamination resulting from eye blinking. Secondly, this study opted for four channels located at the forehead, given their higher cognitive data features and improved conductivity with minimal interference from hair. However, international standards, like the 10–20 system, encompass a broader array of channels, covering the entire scalp. In order to attain higher data relevancy and reliability, it is highly recommended to explore the inclusion of additional channels in future studies. Third, for validation purposes, this study employed a simplified assembly task. In future research, incorporating more complex and practical tasks that closely mimic the actual working processes in workshops and factories would yield more realistic insights. Such an approach could provide a comprehensive understanding of cognitive load variations in real-world work scenarios, contributing to the applicability and relevance of the findings. Lastly, it is important to note that the participants in this study only offer a partial reflection of the broader workforce. Notably, there exists a skewed gender distribution among those who took part in the experiment. Given that gender is a recognized influential factor in shaping human cognitive abilities, this imbalance could significantly influence the outcomes of the study. Consequently, it is strongly advisable to conduct comprehensive, multi-dimensional tests across diverse demographic backgrounds in future research endeavors to ensure a more representative depiction of the broader population. By addressing these limitations in subsequent studies, it will lead to more comprehensive and accurate insights into workers' cognitive workload during assembly tasks and transitions.

## Data Availability

The data that support the findings of this study are available from the corresponding authors upon request.
